# Altered gray matter volume and functional connectivity in medial orbitofrontal cortex of bulimia nervosa patients: A combined VBM and FC study

**DOI:** 10.3389/fpsyt.2022.963092

**Published:** 2022-08-19

**Authors:** Wei-hua Li, Li-rong Tang, Miao Wang, Jia-ni Wang, Ting Guo, Qiong He, Yu-yang He, Zi-ling Lv, Qian Chen, Zheng Wang, Xiao-hong Li, Peng Zhang, Zhan-jiang Li, Zhen-chang Wang

**Affiliations:** ^1^Department of Radiology, Beijing Friendship Hospital, Capital Medical University, Beijing, China; ^2^Beijing Anding Hospital, Capital Medical University, Beijing, China; ^3^The National Clinical Research Center for Mental Disorders and Beijing Key Laboratory of Mental Disorders, Beijing, China; ^4^Chinese Institute for Brain Research, Beijing, China

**Keywords:** bulimia nervosa, eating disorder, voxel-based morphometry, structural MRI, resting-state fMRI

## Abstract

Brain structural and functional abnormalities have been shown to be involved in the neurobiological underpinnings of bulimia nervosa (BN), while the mechanisms underlying this dysregulation are unclear. The main goal of this investigation was to explore the presence of brain structural alterations and relevant functional changes in BN. We hypothesized that BN patients had regional gray matter volume abnormalities and corresponding resting-state functional connectivity (rsFC) changes compared with healthy controls. Thirty-one BN patients and twenty-eight matched healthy controls underwent both high-resolution T1-weighted magnetic resonance imaging (MRI) and resting-state functional MRI. Structural analysis was performed by voxel-based morphometry (VBM), with subsequent rsFC analysis applied by a seed-based, whole-brain voxelwise approach using the abnormal gray matter volume (GMV) region of interest as the seed. Compared with the controls, the BN patients showed increased GMV in the left medial orbitofrontal cortex (mOFC). The BN patients also exhibited significantly increased rsFC between the left mOFC and the right superior occipital gyrus (SOG) and decreased rsFC between the left mOFC and the left precentral gyrus, postcentral gyrus, and supplementary motor area (SMA). Furthermore, the *z* values of rsFC between the left mOFC and right SOG was positively correlated with the Dutch Eating Behavior Questionnaire-external eating scores. Findings from this investigation further suggest that the mOFC plays a crucial role in the neural pathophysiological underpinnings of BN, which may lead to sensorimotor and visual regions reorganization and be related to representations of body image and the drive behind eating behavior. These findings have important implications for understanding neural mechanisms in BN and developing strategies for prevention.

## Introduction

Bulimia nervosa (BN) is an eating disorder with a multidimensional etiology. Its essential feature is recurrent episodes of binge eating combined with a subjective feeling of loss of control, followed by inappropriate compensatory behaviors (such as self-induced vomiting; misuse of laxatives, diuretics, or other medications; fasting; or excessive exercise) to avoid weight gain and overestimation of one's own body size (1). BN usually begins during adolescence in females, and the lifetime prevalence is 2.6%, with an ~10:1 female-to-male ratio (1, 2). Only 30–45% of adults exhibit prolonged remission after receiving the existing forms of treatment, including cognitive behavior therapy and pharmacotherapy (3). The neurobiological mechanisms involved in the development and maintenance of BN remain unclear and have largely hindered the development of therapeutics for this disorder.

Neuroimaging techniques, especially useful in highlighting distinct levels of neuropathology, have been recently applied to investigate the structural and functional abnormalities of the brain in BN, but the conclusions have been inconsistent. Voxel-based morphometry (VBM) is an automated technique and an incredibly powerful tool to assess structural changes in the brain (4). Functional magnetic resonance imaging (fMRI), an inferential neuroimaging technique, can be used to investigate synchronous neural activity to explore functional connectivity (FC) between brain regions (5). Resting-state functional connectivity (rsFC) analysis, measured *via* a stimulus-free fMRI approach, has been used to identify multiple functional networks that are understood to drive behavior (6). A seed-based approach can be used to assess the temporal correlations and neural network reconfiguration between the seed and other regions of the brain (7). For instance, some previous studies based on VBM have shown that, compared with healthy controls, individuals with BN have increased gray matter volume (GMV) in the orbitofrontal gyrus, ventral striatum, insula, precuneus, and paracentral lobules, decreased GMV in the superior temporal gyrus and caudate nucleus, and increased or decreased GMV in the putamen (8–12). Additionally, meta-analyses have revealed that BN patients exhibit altered neural function in brain networks involved in cognitive control, reward processing, affective processing, and visuospatial and body-signal integration, which involve the frontal, parietal, cingulate, ventral striatal, amygdala, insular, somatosensory, and occipital cortices (5, 13–15). It appears that structural changes in brain areas influence cognitive and perceptual function in BN individuals. However, most of these studies have been difficult to replicate with consistent results, and they focus either on structural or functional changes, rather than both (16, 17). In other disease areas, such as anorexia nervosa, migraine and alcohol use disorder, some neurological studies have found coexisting functional and structural differences using whole-brain data-driven approaches (18–20). Furthermore, little is known about how altered regional GMV affects intrinsic brain functional activity in BN individuals. Identifying regional brain structural abnormalities and associated functional alterations may provide specific insights into the developing brain's adaptive or maladaptive changes and reframe our understanding of the pathophysiology underlying BN.

In the present study, using a cross-sectional, neuroimaging imaging approach combining VBM with rsFC analysis to identify specific brain areas related to BN, we aimed to elucidate the underlying structural abnormality and relevant functional changes in patients with BN. We hypothesized that (1) BN patients demonstrate altered GMV in some brain regions, including cognitive control, reward, affect processing, or sensorimotor-related regions; and (2) regions affected in structure also demonstrate neural functional changes. Moreover, the correlation between significant brain regions and clinical features was explored.

## Materials and methods

### Participants

Thirty-one BN patients and twenty-eight age-, sex- and education-matched healthy controls (HCs) were consecutively enrolled in this study. All study subjects were females and right-handed checked by answering questions related to the Edinburgh Handedness Inventory before enrollment (21). The BN patients were recruited from outpatient services of the hospital, and diagnoses were confirmed *via* clinical interviews with a psychiatrist based on the Diagnostic and Statistical Manual of Mental Disorders, 5th Edition (DSM-5) criteria for BN. Patients were required to have been free of any psychotropic medications for at least 2 months before the study. Specific exclusion criteria for all groups were current or past comorbid serious psychiatric disorders, such as bipolar disorder, schizophrenia, major depression and anxiety disorder; with a history of anorexia nervosa or binge eating disorder; alcohol or substance abuse; history of severe head injury, intellectual disability, pregnancy and significant health problems, such as endocrine disorder (including diabetes, hyperthyroidism, hyperlipidemia), cancer, heart, kidney, gastrointestinal, or liver disease; known neurological impairment (e.g., epilepsy). All study subjects had no known MRI incompatibility (e.g., pregnancy, lactation; ferrous implants such as pacemakers and cochlear implants; claustrophobia). Owing to metal denture artifacts in the raw data and abnormal blood biochemical tests, MRI datasets from two BN patients were excluded, with 29 patients and 28 HCs finally used in the analysis.

The present study was approved by the Institutional Review Board of the hospital. Written informed consent was obtained from all participants after providing a full description of the procedure involved. This study was performed in accordance with the ethical standards of the Declaration of Helsinki.

### Clinical variable measures

The demographic questionnaires (including age, gender, education level, weight, and height) of participants were recorded. Before arrival at the scanning visits, all participants were asked to fast for at least 4 h, and upon arrival, they completed the visual analog scale (VAS) to rate their current hunger intensity (0 indicates “not at all”; 10 indicates “extremely”). All participants further completed several self-report assessments, including the Chinese version of the Dutch Eating Behavior Questionnaire (DEBQ) (22), Eating Disorder Inventory-I (EDI-I) (23), Eating Attitudes Test (EAT-26) (24), Beck Depression Inventory (BDI) (25), and self-rating anxiety scale (SAS) (26). The DEBQ, used to assess eating behavior, consists of 33 items in three subscales reflecting restraint eating (overeating in the wake of attempted restraint), emotional eating (eating in response to emotional distress), and external eating (eating in response to external food cues). Each statement in the DEBQ was rated on a 5-point Likert scale (ranging from 1 “never” to 5 “very often”). In addition, the EDI and EAT-26 were applied to evaluate symptoms and the characteristic features of eating disorders, while depression and anxiety status were measured by the BDI-II and SAS, respectively. The Chinese version of the above scales has good reliability and validity and is applicable among Chinese adults (22–25, 27).

### MRI data acquisition

All MRI images were collected on a 3.0 T MRI system (Prisma, Siemens, Erlangen, Germany) equipped with a 64-channel phase-array head coil. A conventional brain axial T2 sequence was obtained to exclude any possible abnormality in the brain. High-resolution anatomical T1-weighted images were acquired in sagittal orientation using a 3D magnetization-prepared rapid gradient-echo (MP-RAGE) sequence. The scanning parameters were as follows: repetition time (TR) = 2,530 ms; echo time (TE) = 2.98 ms; inversion time (TI) = 1,100 ms; flip angle (FA) = 7°; 192 sagittal slices with slice thickness = 1 mm and interslice gap = 1 mm; bandwidth = 240 Hz/Px; field of view (FOV), 256 × 256 mm^2^; data matrix, 256 × 256; and isotropic voxel size, 1 × 1 × 1 mm^3^. Blood oxygen level-dependent (BOLD) resting-state functional images were obtained with an echo-planar imaging sequence with the following parameters: TR = 2,000 ms; TE = 30 ms; FA, 90°; number of slices = 33; slice thickness = 3.5 mm; interslice gap = 1 mm; bandwidth = 2,368 Hz/Px; FOV = 224 × 224 mm^2^; data matrix = 64 × 64; and total volumes, 240.

During the entire scanning process, which lasted ~14 min, we used tight but comfortable foam padding around the subject's head to minimize head motion and earplugs to reduce imaging noise. All participants were asked to close their eyes, stay awake, breathe evenly, and try to avoid specific thoughts. After each sequence, all subjects reported by conversation that they had not fallen asleep during the imaging protocol.

### Data analysis

#### Clinical data analysis

The baseline demographic information and clinical characteristics were analyzed using SPSS 25.0 software (SPSS Inc., Chicago, IL). Between-group comparisons were performed using independent two-sample *t*-tests, as appropriate. A *p* < 0.05 was considered statistically significant. Continuous variables are presented as the mean with 95% confidence intervals (CIs).

#### VBM analysis

Structural data preprocessing and statistical analyses were performed using the Computational Anatomy Toolbox 12 (CAT12: http://dbm.neuro.uni-jena.de/cat/) and Statistical Parametric Mapping 12 (SPM12: http://www.fil.ion.ucl.ac.uk/spm) in the MATLAB environment. First, each participant's anatomical images were reoriented to have the same spatial orientation by manually setting the anterior commissure as the origin. Then, the standard preprocessing procedure was performed using the Segment Data module in the CAT12 manual. The preprocessing included (1) estimation of a non-linear deformation field that best overlaid the tissue probability maps on the individual subject's images; (2) segmentation of the images into gray matter (GM), white matter (WM), and cerebrospinal fluid (CSF) to calculate the overall tissue volume (GM, WM, and CSF volume) and total intracranial volume (TIV) in the native space; (3) normalization of the GM segments to the Montreal Neurological Institute (MNI) template using the high-dimensional diffeomorphic anatomic registration through the exponentiated lie algebra (DARTEL) method with a voxel size of 1.5 × 1.5 × 1.5 mm^3^; (4) modulation by the “non-linear only” components derived from spatial normalization; (5) bias-field correction to remove intensity non-uniformities; and (6) smoothing using a Gaussian kernel with a 6-mm full-width at half maximum (FWHM) Gaussian filter to create a local weighted average of the surrounding voxels. After completing these image analyses, we obtained smoothed and modulated GM images to be used for statistical analysis. Two-sample *t*-tests were used to assess group differences while incorporating age, education level, and BDI scores, and TIV as covariates. The statistical threshold criterion was set with a preliminary uncorrected threshold of p < 0.001. The cluster was corrected for multiple comparisons with family wise error (FWE, *p* < 0.05).

#### Seed-to-voxel resting-state functional connectivity analysis

To further investigate possible correlations of regional GMV abnormalities with other brain regions in the BN patients, rsFC analysis was carried out using a seed-based, whole-brain voxelwise approach and defining the brain regions with abnormal GMV as a seed. Preprocessing was performed using SPM12 and the Resting-State fMRI Data Analysis Toolkit (RESTplus: http://www.restfmri.net). In brief, preprocessing procedures involved discarding the first 10 functional volumes (the first 20 s of fMRI data), slice timing, head motion correction, normalization with resampling voxel size at 3.0 mm × 3.0 mm × 3.0 mm voxels, smoothing using a 6.0-mm FWHM Gaussian kernel, detrending, nuisance covariate regression (global signal, WM signal, and CSF signal), and bandpass filtering (0.01–0.08 Hz) for each voxel. No subjects were excluded based on the correction exclusion criteria (spatial movement in any direction of more than 2.0 mm or 2 degrees). Further rsFC analyses were conducted using RESTplus software. The averaged time series for the seed was calculated for the reference time course. Cross-correlation analyses were performed between the mean time course in the seed and the time series of other voxels in the whole brain. To improve normality, the correlation coefficient maps were then converted into Fisher-*z* maps by bivariate Fisher's *z*-transform. Two-sample *t*-tests were used to compare rsFC differences between the BN patients and HCs by adding age, education level, body mass index (BMI), TIV, and BDI scores as covariates to exclude the effects of confounding factors. The statistical comparisons were first thresholded on the voxel level at *p* < 0.001 (uncorrected) and corrected for multiple comparisons by applying Gaussian random field theory (GRF, *p* < 0.05).

#### Correlation analyses

To examine the relationship between rsFC strength and the clinical data, we extracted the average *z* scores from all surviving clusters. Pearson's correlations were conducted in the BN group to examine the relations between rsFC *z* scores within each of the clusters and the clinical data, including disease duration, binge eating frequency, BMI, and DEBQ, EDI-II, and EAT-26 scores. Significant correlations were determined according to *p* < 0.05 (false discovery rate, FDR-corrected).

In addition, we further tested whether a correlation existed between VBM findings and the potential correlations results in the BN group through a standard three-variable mediation analysis which performed with the R package Mediation (version 4.5.0) (26). A bootstrap strategy with 10,000 resampling iterations was used to estimate the bias-corrected significance of the mediation. *P* < 0.05 is considered statistically significant.

## Results

### Baseline characteristics

As shown in Table 1, the BN patients and HCs showed no significant differences in demographic or clinical data, including age, BMI, years of education, and fasting hours. The BN patients had higher DEBQ, EDI-BN, EAT-26, BDI, and SAS scores than the HCs (*p* < 0.05).

**Table 1:** Baseline demographics and clinical characteristics of participants.

**Characteristics**	**Bulimia nervosa patients (*****n*** = **29)**		**Healthy Controls (*****n*** = **28)**		***P***
	**Mean**	**SD**	**Mean**	**SD**	
Age (years)	24.00	4.85	25.68	2.75	0.113
BMI (kg/m^2^)	20.85	3.49	21.12	2.15	0.731
Age of illness onset (years)	20.44	4.93	–	–	–
Illness duration (months)	38.04	41.47	–	–	–
Binge eating per week (times/week)	6.37	4.79	–	–	–
Education (years)	16.31	2.12	16.82	2.06	0.360
Fasting hours (h)	9.52	3.33	8.66	4.11	0.391
DEBQ–total	121.41	16.44	87.04	18.95	0.000^*^
DEBQ–restrained eating	37.59	6.85	28.86	7.89	0.000^*^
DEBQ–emotional eating	48.34	10.99	27.14	10.77	0.000^*^
DEBQ–external eating	35.14	4.67	31.04	5.80	0.015^*^
EDI-BN	34.90	5.31	12.04	3.71	0.000^*^
EAT	42.93	11.64	13.50	10.25	0.000^*^
BDI	23.97	11.13	3.43	2.78	0.000^*^
SAS	54.91	12.35	33.30	5.62	0.000^*^

BMI, Body Mass Index; SD, standard derivative; DEBQ, Dutch Eating Behavior Questionnaire; EDI, Eating Disorder Inventory; EAT, Eating Attitudes Test; BDI, Beck Depression Inventory; SAS, Self-rating Anxiety Scale.

* p < 0.05 is considered statistically significant.

### VBM results

**Figure 1:**
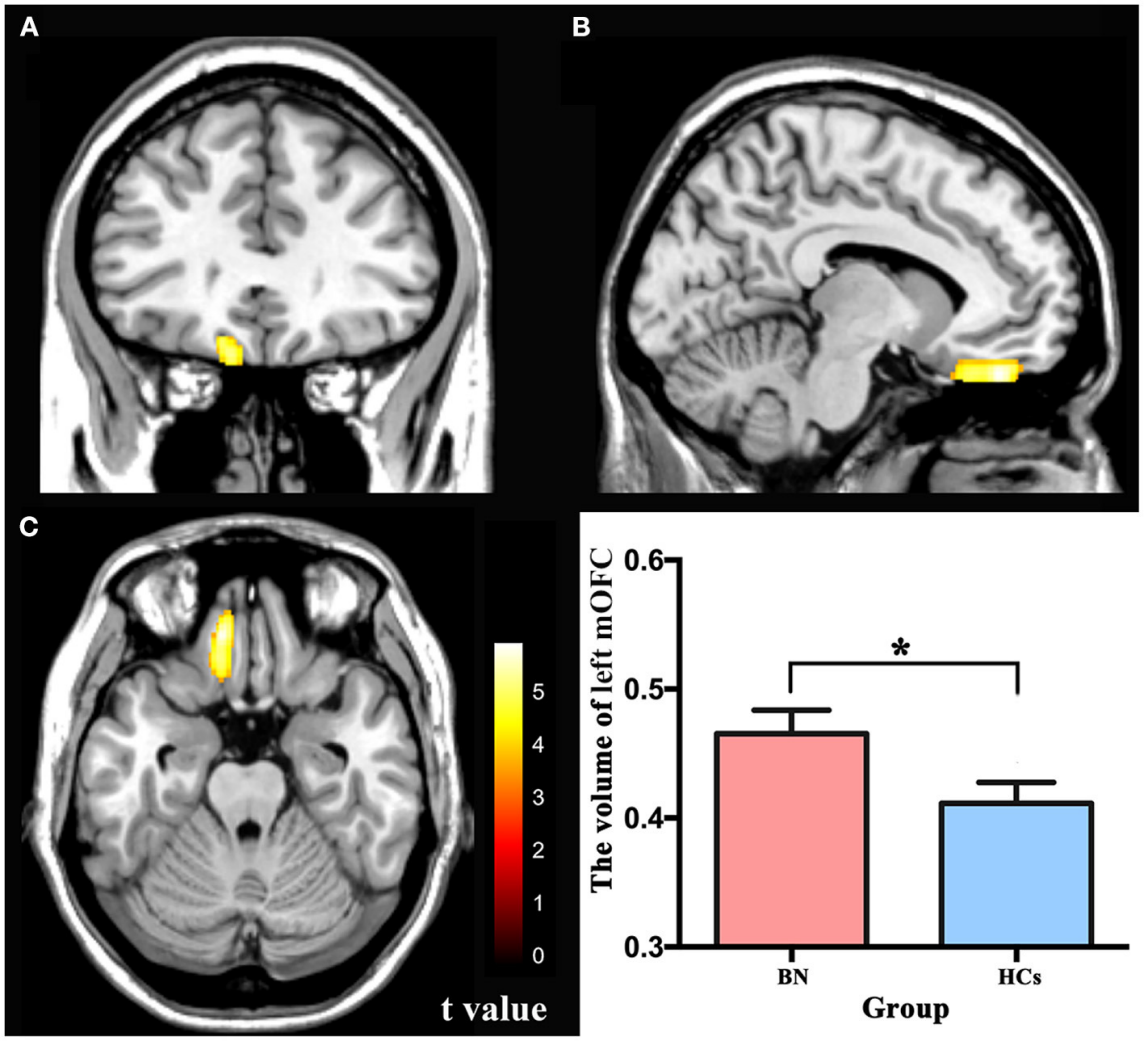
**(A–C)** The yellow region indicates larger GMV of the left mOFC in the bulimia nervosa patients than in the healthy controls (voxel-level *p* < 0.001 uncorrected and cluster-level *p* < 0.05 FWE-corrected). GMV, gray matter volume; mOFC, medial orbitofrontal cortex; FWE, family wise error. * *p* < 0.05 (FWE-corrected).

There were no significant differences between the BN patients and HCs in the total GMV, WM volume, CSF, or TIV, which is consistent with some previous studies (16). Compared with the HCs, the BN patients showed increased volume in the left medial orbitofrontal cortex (mOFC) (FWE corrected) (Table 2; Figure 1). Compared with the HCs, the BN patients did not have decreased volume in any brain region.

**Table 2:** Regional GMV abnormalities between bulimia nervosa patients and healthy controls.

**Brain regions**	**Hemisphere**	**Cluster size (voxels)**	**Peak t values**	**MNI coordinates**		
				**X**	**y**	**Z**
**BN** **>** **HC**
mOFC	L	609	5.41	−9	40.5	−24

GMV, gray matter volume; MNI, Montreal Neurological Institute; BN, bulimia nervosa; HC, healthy controls; mOFC, medial orbitofrontal cortex; L, left.

### RsFC patterns in participants with BN

**Figure 2:**
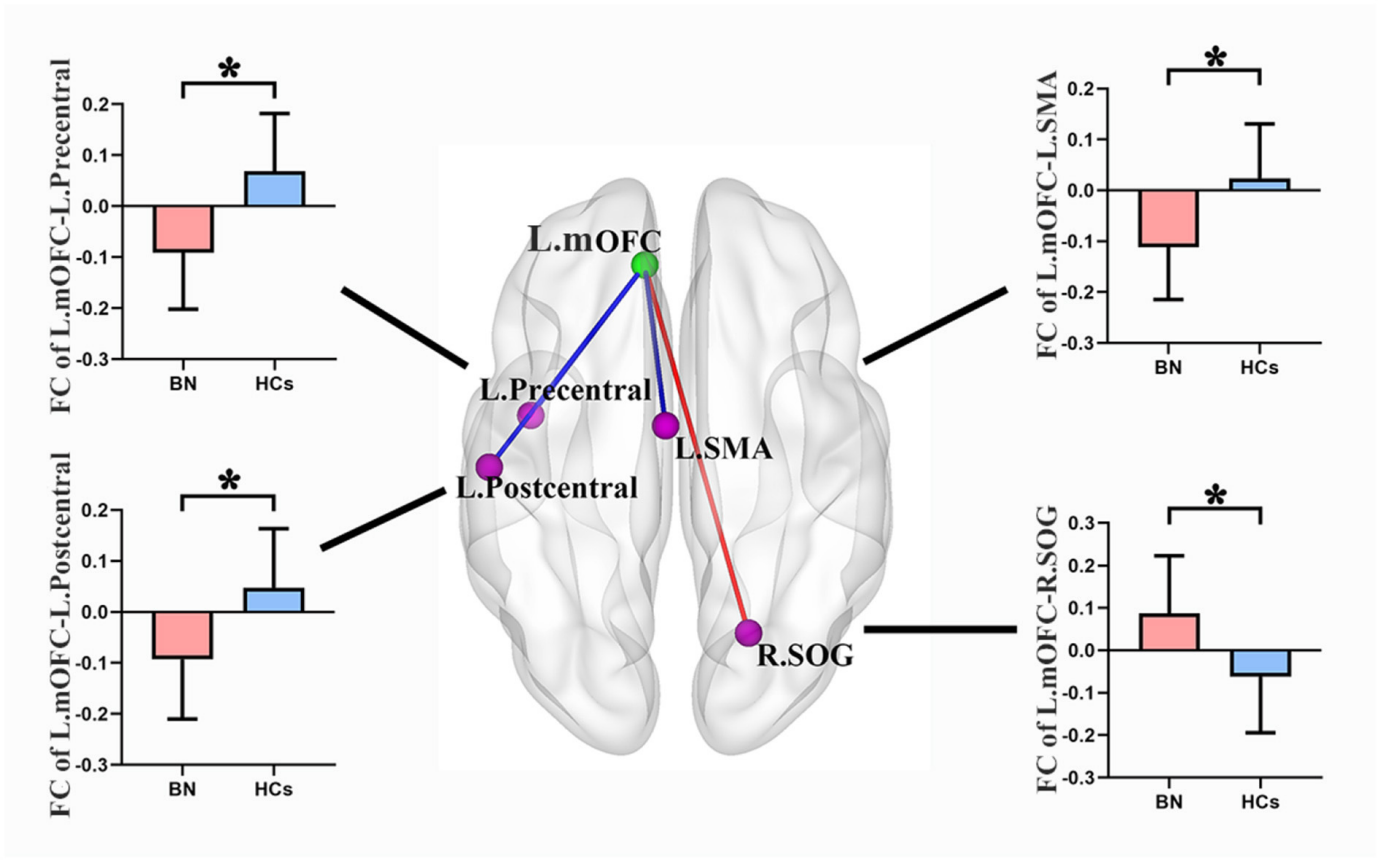
Compared with healthy controls, the bulimia nervosa group showed significantly increased rsFC between the left mOFC and the rSOG, but decreased rsFC between the left mOFC and the left precentral gyrus, left postcentral gyrus and left SMA (voxel-level *P* < 0.001 uncorrected and cluster-level *P* < 0.05 GRF correction). Nodes are color-coded with the seed region (green) and the rsFC differential region (purple). The rsFC between nodes are represented by lines, with red indicating increased and blue indicating decreased. rsFC, resting-state functional connectivity; mOFC, medial orbitofrontal cortex; SOG, superior occipital gyrus; SMA, supplemental motor area; GRF, gaussian random field theory. * *p* < 0.05 (GRF-corrected).

Compared with the HCs, the BN patients showed increased rsFC between the left mOFC and right superior occipital gyrus (rSOG) and decreased rsFC between the left mOFC and the left precentral gyrus, left postcentral gyrus, and left supplemental motor area (SMA) (GRF corrected) (Table 3; Figure 2).

**Table 3:** Altered rsFC from the left mOFC to other brain regions in bulimia nervosa patients.

**Brain regions**	**Hemisphere**	**Cluster size (voxels)**	**Peak t values**	**MNI coordinates**		
				**x**	**y**	**Z**
Precentral	L	79	−4.832	−42	−3	9
Postcentral	L	65	−4.336	−54	−18	27
SMA	L	67	−5.175	−3	−6	51
SOG	R	48	4.206	21	−66	30

rsFC, resting-state functional connectivity; mOFC, medial orbitofrontal cortex; MNI, Montreal Neurological Institute; L, left; R, right; SMA, supplemental motor area; SOG, superior occipital gyrus.

### Correlations between DEBQ scores and RsFC values

**Figure 3:**
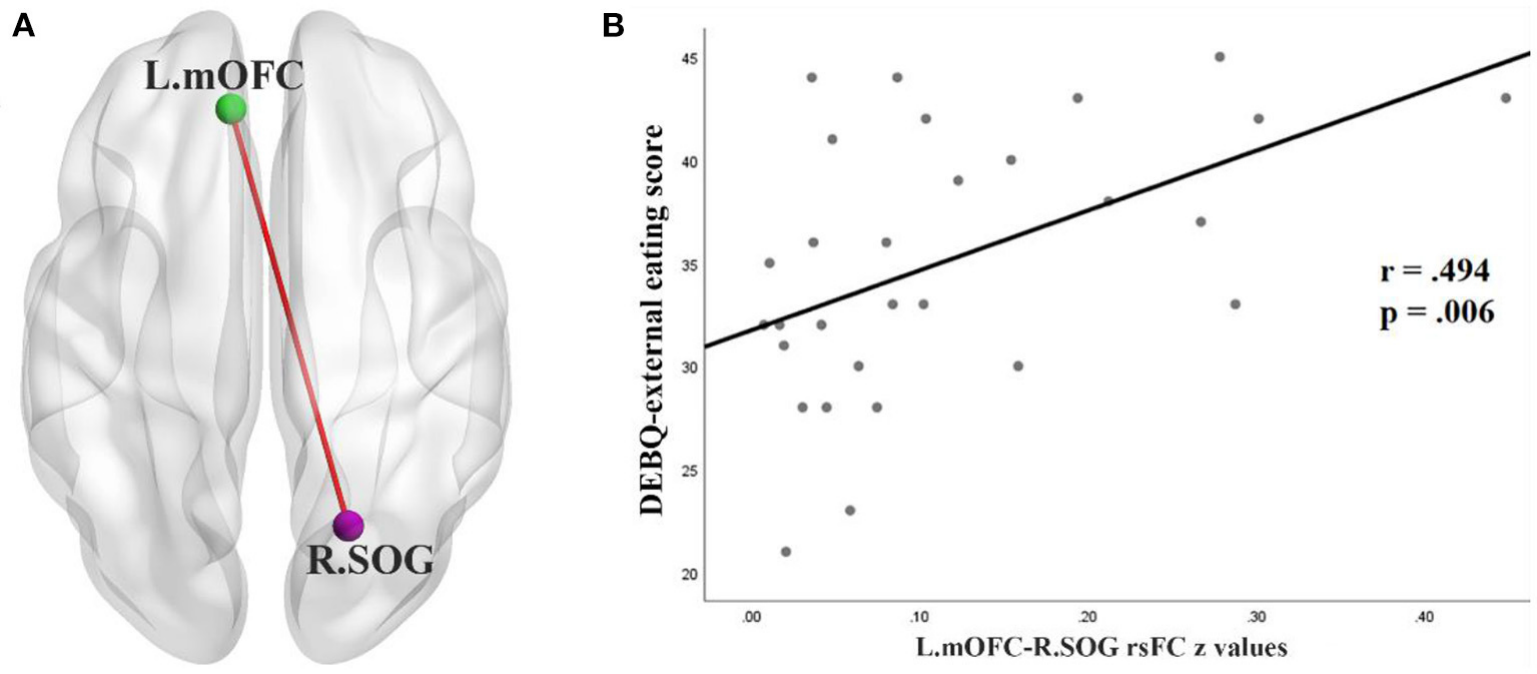
**(A)** rsFC between the left OFC (L.mOFC) and right SOG (R.SOG) in patients with BN. **(B)** Correlation in the BN patients between DEBQ-external eating scores and rsFC values for the L.mOFC—R.SOG (*p* < 0.05 corrected with FDR). rsFC, resting-state functional connectivity; mOFC, medial orbitofrontal cortex; SOG, superior occipital gyrus; DEBQ, Dutch Eating Behavior Questionnaire; FDR, false discovery rate.

In the BN group, rsFC *z* values between the left mOFC and rSOG was positively correlated with DEBQ-external eating scores (*r* = 0.494, *p* < 0.05) (FDR corrected) (Figure 3). No additional significant correlations were found.

**Figure 4:**
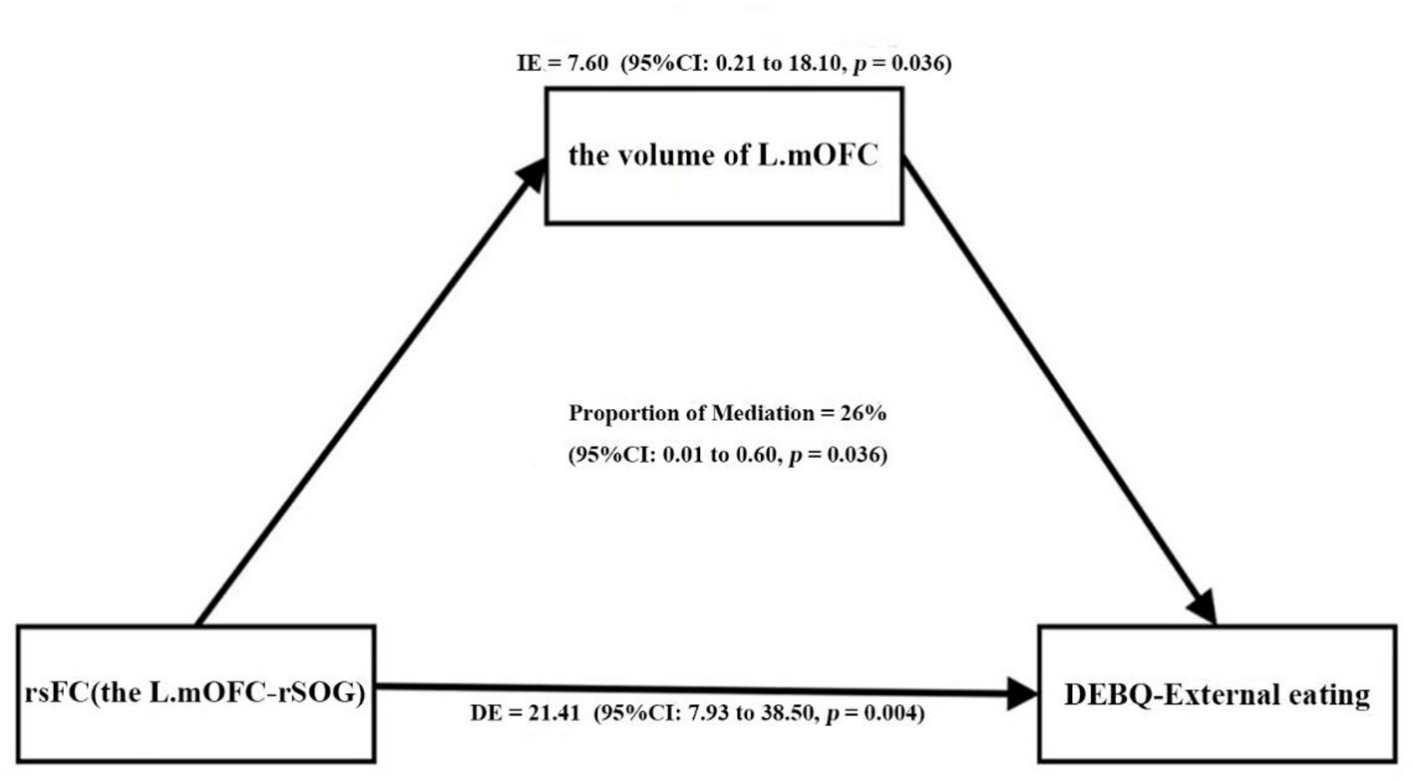
The volume of the left mOFC partially mediated the relationship between rsFC (the left mOFC—rSOG) and DEBQ-external eating (Prop.mediated = 26.2%, *p* = 0.036). mOFC, medial orbitofrontal cortex; rsFC, resting-state functional connectivity; SOG, superior occipital gyrus; DEBQ, Dutch Eating Behavior Questionnaire.

Furthermore, as shown in Table 4 and Figure 4, the *p*-value of the mediation variable is 0.036 (*p* < 0.05), indicating that the volume of left mOFC partially mediated the relationship between rsFC (the left mOFC—rSOG) and DEBQ-external eating and the mediating effect accounted for 26.2%.

**Table 4:** Mediation analysis for the volume of left mOFC and the relationship between rsFC (the left mOFC-rSOG) and DEBQ-external eating.

	**Estimate**	**95% CI Lower**	**95% CI Upper**	***P*-value**
Indirect effect	7.60	0.21	18.10	0.036^*^
Direct effect	21.4	7.9310	38.50	0.004^*^
Total effect	29.0	14.3	48.3	0.000^*^
Prop. Mediated	0.2620	0.01	0.6	0.036^*^

mOFC, medial orbitofrontal cortex; rsFC, resting-state functional connectivity; SOG, superior occipital gyrus; DEBQ, Dutch Eating Behavior Questionnaire.

* P < 0.05 is considered statistically significant.

## Discussion

In our study, we found that relative to HCs, BN patients had increased GMV in the left mOFC. Furthermore, in BN patients, the structurally altered left mOFC had increased rsFC with the rSOG but decreased rsFC with the left precentral gyrus, postcentral gyrus and SMA. Consistent with our hypothesis, we found that the mOFC in BN patients showed local structural and neural functional network reorganization. In addition, the correlation analysis showed that the rsFC *z* values between the left mOFC and rSOG was correlated with DEBQ-external eating scores in the BN group and the volume of left mOFC partially mediated the relationship between rsFC (the left mOFC—rSOG) and DEBQ-external eating, which may provide insight into how neural abnormalities are related to the symptomatology of BN. This structural and functional neuroimaging study could further support the involvement of the mOFC in the pathophysiology of BN.

The OFC, occupying the ventral surface of frontal lobes, plays a crucial role in some complex human behaviors, such as food valuation, social cognition, affect regulation, and reward-based decision making (28, 29). Our results showed that the BN patients had structural brain alterations in the left mOFC compared with the HCs. This finding is in line with a previous VBM study that also reported an increased volume in the mOFC in BN patients relative to HCs and suggests that this structural abnormality might be related to food reward processing and/or self-regulation dysfunction (8). A recent review summarized that the OFC plays a relevant role in food intake control and satiety, and its increased volume in eating disorders could possibly drive food avoidance through early satiation and/or disturbed reward valuation of food stimuli (30). The main brain reward circuit includes the ventral tegmental area (VTA) and the nucleus accumbens (NAc), which is part of the striatum. Brain reward system dysfunction in BN has been widely reported. The OFC, anterior cingulate cortex (ACC), and ventromedial prefrontal cortex (PFC) are involved in high-order cognitive processes within the reward circuitry and are necessary for reward processing, inhibition of emotional responses, and habit formation, and they promote behavioral outcomes (30). Additionally, the OFC plays a key role in hedonic and motivational aspects of reward and is integral in controlling behaviors associated with reward and punishment (31). In addition, a large number of clinical psychology studies have found that BN patients have obvious impulsivity, which is manifested by decreased decision-making abilities, impaired inhibitory functions, and other behavioral characteristics (32, 33). Additionally, a systematic review showed that dysfunction in frontostriatal circuits may be associated with recurrent binge-eating and purging behaviors in BN patients (34). Previous studies have found that the OFC is also associated with impulsivity, which helps to explain the uncontrolled abnormal eating behaviors in individuals with BN (35, 36). Additionally, an event-related fMRI study demonstrated that diminished frontostriatal brain activation in BN patients contributed to the severity of recurrent binge-eating characteristics (37). The increased volume of the mOFC may be related to diminished frontostriatal brain activation. More recently, some studies have suggested that the OFC is a key node of the cognitive control neurocircuitry, more specifically, the inhibition system, and may be involved in affective processing (5, 13, 29). Thus, we further confirmed a crucial role of the mOFC in BN and speculate that this regional structural alteration may be related to the pathogenesis and maintenance of disease. To investigate the regulatory relationship in the mOFC, it is necessary to apply additional rsFC analyses to evaluate the features of regulation patterns in this brain region.

In accordance with our second hypothesis, we observed decreased functional connections between the left mOFC and left sensorimotor cortex but increased connections between the left mOFC and right visual occipital areas. The motor cortex, including the primary motor cortex, premotor cortex, and SMA, is involved in motor planning, preparation, and control of motor execution, including behavioral inhibition (38). The close connection between the sensorimotor cortex and reward-related regions has been widely reported in obesity-related studies (31). Such an uncoupling between the left mOFC and the left motor cortex in BN patients might drive uncontrolled binge eating and inappropriate purging behaviors. Conversely, one of the diagnostic criteria for BN is a distorted representation of one's own body (1). Patients with BN are characterized by fear of fatness and excessive concern with body shape and weight. Several studies have suggested that the somatosensory and occipitotemporal systems are involved in body image perception (39, 40). A study by Lavagnino et al. demonstrated that BN patients show reduced rsFC within the somatosensory network and between the paracentral lobule and the right middle occipital gyrus, which are relevant to body image (41). Another study of resting-state whole-brain FC of striatal subregions in BN found a decreased rsFC in primary sensorimotor and occipital areas for nearly all striatal subregions and observed significant correlations between rsFC of the striatum and somatosensory/occipital areas with the severity of bulimia (17). These previous studies partially overlap with our results, while our study links the rsFC changes in the sensorimotor and visual occipital regions to the mOFC. However, we observed increased rsFC in the left mOFC and rSOG. A correlation between the increased rsFC values and external eating behavior scores was also found in this study. A systematic review summarized that restrained eaters show an over responsiveness to external food-relevant cues, which reflects an incompatibility between sensitivity to eating enjoyment and the goal of eating control (42). Visual processing is an important input interface to reward circuitry. Accordingly, when exposed to food-relevant stimuli primes, BN patients might show that priming with eating rewards decreases accessibility to eating control. This proposal is further suggested by the significant positive correlation observed between rsFC of the left mOFC and right SOG with the external eating behavior scores. Furthermore, in the BN group, the increased left mOFC volume played a mediating role in this significant positive correlation to some extent, which may support a correlation between structure and function of this region. This result provided further evidence for the involvement of mOFC in regulating external eating behavior of BN. Taken together, we found that neural functional network reorganization and structural changes in the mOFC, which may be involved in modulating visuospatial perception and driving abnormal eating behavior in BN.

The present investigation has the following limitations. First, the present study is limited to a modest sample size. We included only adult women with BN, so the results cannot be generalized to men. However, BN patients were carefully selected to exclude confounding effects of comorbidities and medication use. Second, in this study, only one brain region was selected as a seed based on the VBM analysis, potentially explaining the limited number of significantly altered FCs observed. Third, this was a descriptive, not a mechanistic study. Finally, we did not control for menstrual status, which may also affect attention-related neural functioning (43). Thus, future multimodal research should involve a larger sample size with more stringent control of confounding factors to explore the abnormal structural features and functional activation in BN.

## Conclusion

Local structural alterations and neural functional network reorganization in the mOFC suggest that this structure is involved in the neural pathophysiological underpinnings of BN. We found that neural functional network reorganization in the mOFC involves the sensorimotor and visual regions that mediate body image and drive eating behavior, which can provide new insight into how neural abnormalities are related to the symptomatology associated with BN. ‘

## Data availability statement

The raw data supporting the conclusions of this article will be made available by the authors, without undue reservation.

## Ethics statement

The studies involving human participants were reviewed and approved by the Institutional Review Board of the Beijing Friendship hospital, Capital Medical University. The patients/participants provided their written informed consent to participate in this study.

## Author contributions

W-hL, L-rT, and PZ conceptualized and designed the study. W-hL was responsible for conducting the analyses, preparing the first draft of the manuscript, and preparing the manuscript for submission. L-rT, TG, Y-yH, and Z-lL participated in the collection of enrolled BN patients and clinical data, supervising the analyses, and editing drafts of the manuscript. J-nW, QC, ZW, and MW completed the participants' MRI data collection and initial data preprocessing. X-hL, Z-cW, Z-jL, and PZ reviewed and edited the manuscript and approved the final version. All the authors contributed substantially to the writing and revising of the manuscript, approved it for publication.

## Funding

This work was supported by the National Natural Science Foundation of China (Grant Numbers: 82001790), the Beijing Hospitals Authority Youth Programme (Grant Numbers: QML20191902), the seed project from Beijing Friendship Hospital, Capital Medical University (Grant Numbers: YYZZ201917), Beijing Scholar 2015 (Zhenchang Wang), Beijing key Clinical Discipline Funding (No. 2021-135), and Beijing Key Laboratory for Mental Disorders (2021JSJB03).

## Conflict of interest

The authors declare that the research was conducted in the absence of any commercial or financial relationships that could be construed as a potential conflict of interest.

## Publisher's note

All claims expressed in this article are solely those of the authors and do not necessarily represent those of their affiliated organizations, or those of the publisher, the editors and the reviewers. Any product that may be evaluated in this article, or claim that may be made by its manufacturer, is not guaranteed or endorsed by the publisher.
